# Inherent and Acquired Resistance to Paclitaxel in Hepatocellular Carcinoma: Molecular Events Involved

**DOI:** 10.1371/journal.pone.0061524

**Published:** 2013-04-16

**Authors:** Avtar Singh Meena, Aanchal Sharma, Ratna Kumari, Naoshad Mohammad, Shivendra Vikram Singh, Manoj Kumar Bhat

**Affiliations:** National Centre for Cell Science, Ganeshkhind, Pune, India; University of Navarra School of Medicine and Center for Applied Medical Research (CIMA), Spain

## Abstract

Hepatocellular carcinoma (HCC) is a primary malignancy of the liver and is a major cause of cancer related deaths worldwide. Only 10 to 20% of HCC can be surgically excised. Therefore, chemotherapeutic intervention and treatment is essential for achieving favorable prognosis. However, therapeutic outcome of chemotherapy is generally poor owing to inherent resistance of cancer cells to the treatment or due to development of acquired resistance. To differentiate and delineate the molecular events, we developed drug resistant Hep3B cells (DRC) by treating cells with the increasing concentration of paclitaxel. We also developed a unique single cell clone of Hep3B cells (SCC) by selecting single cell colonies and screening them for resistant phenotype. Interestingly, both DRC and SCC were resistant to paclitaxel in comparison to parental Hep3B cells. We analyzed the contributory factors that may be involved in the development of resistance. As expected, level of P-glycoprotein (P-gp) was elevated in DRC. In addition, Caveolin-1 (Cav-1), Fatty acid synthase (FASN) and Cytochrome P450 (CYP450) protein levels were elevated in DRC whereas in SCC, FASN and CYP450 levels were elevated. Downregulation of these molecules by respective siRNAs and/or by specific pharmacological inhibitors resensitized cells to paclitaxel. Interestingly, these drug resistant cells were also less sensitive to vinblastine, doxorubicin and methotrexate with the exception of cisplatin. Our results suggested that differential levels of P-gp, Cav-1 and FASN play a major role in acquired resistant phenotype whereas FASN level was associated with the presentation of inherent resistant phenotype in HCC.

## Introduction

Hepatocellular carcinoma (HCC) is widespread primary malignant tumor of the liver. It is the third and ninth most common cause of cancer associated deaths in men and women, respectively [1], [2]. Surgery is the only appropriate treatment for HCC, however, only 10 to 20% of HCC can be surgically excised. Therefore, for the larger part of HCC, chemotherapy remains to be the alternative treatment strategy though with insignificant benefits [3]. The diminished effectiveness of chemotherapy is either because of inherent resistance properties or due to acquired resistance. Cancer cells exhibit inherent resistant phenotype because of certain genetic alteration or they acquire resistance following exposure to drug(s) [4], [5], [6], [7].

Development of resistance has been attributed to altered transport of drugs across the plasma membrane, enhanced DNA repair mechanisms, alteration and non-functionality of the target molecules, genetic responses to growth factors and diminished/limited access to target cells [8]. Because of these, chemotherapy has turned out to be increasingly complicated and therefore the anticipated success rate is much below than expected. Also, question remains to be answered as to why degree of resistance varies between the individuals and also for the drugs utilized.

Paclitaxel is utilized for treating non-small cell lung cancers, ovarian, prostate, head and neck, bladder, esophageal cancers, prostate tumors, gastric carcinoma, adrenocortical carcinoma, leukemia, human glioma and hepatocellular carcinoma [9], [10], [11]. It stabilizes microtubule structure by inhibiting their assembly and disassembly and blocking cells in G2/M phase.

Cell membrane being the primary entry point for the drugs, it is likely that membrane associated proteins play a major role in the development of resistance, though multiple mechanisms may contribute to this phenomenon. One such protein, Cav-1 is a major component of specialized plasma membrane micro domain called as caveolae and it has been reported to play a crucial role in signal transduction, gene regulation and protein translocation. Reports highlight the important roles of Cav-1 in tumor progression, metastasis, invasion and cell survival [12], [13], [14], [15]. Also elevated levels of fatty acid synthase (FASN) have been associated with the development of resistance towards doxorubicin in breast cancer cells [16]. Till now, no report details the factors involved in the development of inherent and acquired drug resistance in HCC. Thus, an attempt was made to investigate the factors involved in the development of drug resistance, especially, the role of membrane as well as other cellular proteins.

To achieve this goal, we developed acquired drug resistant Hep3B cells (DRC) and an inherent drug resistant single cell clone of Hep3B cells (SCC) which exhibited resistance to paclitaxel. We report that Cav-1, P-gp, CYP450 and FASN levels were enhanced in DRC, whereas in SCC only FASN and CYP450 levels were increased in comparison with the parental cells. Using specific siRNAs and inhibitors, we demonstrate that apart from P-gp, Cav-1 levels are also associated with acquired resistance in DRC. In inherently resistant SCC, the level of FASN appears to contribute towards resistance phenotype. This work highlights the association of distinct mechanisms in conferring intrinsic and acquired resistance and suggests the cellular proteins those may have to be probed into for combating drug resistance in cancer cells.

## Materials and Methods

### Cells and Culture Conditions

Hep3B, HepG2 and SK-HEP-1 (Hepatocellular Carcinoma) cells, were purchased from American Type Culture Collection (ATCC; Manassas, VA, USA) and maintained in our in-house cell repository at National Centre for Cell Science (NCCS), Pune, India. HepG2, SK-HEP-1, parental Hep3B cells, acquired drug resistant Hep3B cells (DRC) and inherently drug resistant clone from Hep3B cells (SCC) were routinely cultured in Dulbeccòs modified Eagles medium (DMEM) supplemented with 10% heat inactivated fetal bovine serum (Hyclone, UT, USA), penicillin (100 U/ml) and streptomycin (100 µg/ml) (Invitrogen Corporation, CA, USA) at 37°C with 5% CO_2._ Only DRC were routinely cultured in above mentioned media along with 10 nM paclitaxel.

### Development of Drug Resistant Clones

For development of acquired drug resistant cells (DRC), Hep3B cells were exposed to increasing concentration of paclitaxel ranging from 0.3 to 1 µM in complete medium. Briefly, Hep3B cells were seeded in 60 mm petri plates and allowed to grow. After 24 h incubation, paclitaxel (300 nM) was added for another 48 h. Subsequently, medium was removed and fresh, drug free medium was added. The cells were incubated and allowed to grow. When confluency was reached, cells were trypsizined, replated and these cells were re-exposed to double the dose of drug. This process was repeated until clones developed resistance to 1 µM paclitaxel. After prolonged (>3 months) exposure to increasing concentration of paclitaxel, surviving clones were pooled together and termed as drug resistant cells (DRC) and these were used for subsequent experiments. For development of single cell clone (SCC), 1×10^3^ Hep3B cells were seeded in a 100 mm petri plate and allowed to form single cell clones. Individual clones were expanded and screened for resistance towards paclitaxel. The clone No.6 exhibiting resistance towards paclitaxel was used for further experiments and termed as single cell clone (SCC).

### Drugs, Chemicals and Antibodies

Paclitaxel, methyl β-cyclodextrin (MCD) and methylthiazoletetrazolium (MTT) were purchased from Sigma-Aldrich (Sigma Aldrich, MO, USA). Cerulenin was purchased from Calbiochem (Calbiochem, CA, USA). Paclitaxel and cerulenin were dissolved in ethanol and MCD was dissolved in water. Working solutions were diluted in culture medium immediately before use. siRNA specific for Cav-1, CYP450 and FASN and antibodies against Cav-1, FASN, CYP450, P-gp, Hsp70, Hsp90, HSP40, Hsp60, Hsp27 and β-tubulin were purchased from Santa Cruz Biotechnology (Santa Cruz, CA, USA). In all experiments involving inhibitors, cells were pre-treated with indicated concentrations of inhibitor 1 h prior to the addition of paclitaxel and continuously exposed to the inhibitors for duration of treatment. Only exception was MCD, where pre-treatment was given for 4 h and thereafter medium was replaced with fresh medium without MCD. Radiolabeled [^3^H(G)] paclitaxel uptake and MTT assay was done as described previously [17].

### siRNA Transfection

Hep3B cells, DRC and SCC were seeded and transfected with respective siRNA as described by Chhipa *et al* [18]. Cells were further grown for 24 h followed by addition of 300 nM paclitaxel for another 48 h. MTT assay was performed or whole cell lysates were prepared for immunoblotting as described previously [17], [18].

### Long Term Survival Assay

Approximately 1×10^3^ Hep3B cells, DRC and SCC were plated in 12 well plates. Next day, these cells were treated with inhibitors or transfected with siRNA as per the experimental requirements. After 24 h treatment with inhibitor or transfection with siRNA, medium was removed and fresh medium containing 300 nM paclitaxel was added for additional 48 h. Thereafter drug containing medium was replaced with fresh medium without paclitaxel. Cells were allowed to grow for ∼21 days wherein medium was changed every fourth day. Subsequently, staining was performed as described previously [17].

### Morphological Examination of Paclitaxel-resistant Cells

Hep3B cells, DRC and SCC (5×10^5^) were seeded in 35 mm petri plates. After 24 h incubation, cells were treated with 1 µM paclitaxel for 48 h and untreated cells served as control. The cells were washed twice with DMEM, the morphology of cells was visualized and photographed using vertical microscope (Olympus, Tokyo, Japan).

### Flow Cytometry for Cell Cycle Analysis

Cells (5×10^5^) were plated in 35 mm petri plates, allowed to adhere for 24 h followed by harvesting of cells by trypsinization and processed for flow cytometry as described by Chhipa *et al* [18].

### Cellular Protein Lysate Preparation and Immunoblotting

Whole cell lysate were prepared and western blotting was performed as described previously [18]. Wherever required, blots were stripped and reprobed with desired antibodies. Otherwise, gels were run in duplicate and probed for desired proteins and compiled.

### Rhodamine Efflux Assay

To measure efflux of Rhodamine-123 (Rh-123), which reflects P-gp transport activity, Hep3B cells, DRC and SCC were seeded in 60 mm petri plates and allowed to adhere for 24 h. Cells were washed thrice with phosphate buffer saline (PBS) and incubated for 30 min at 37°C in PBS containing 2 µM of Rhodamine-123 (Rh-123) with or without verapamil. Rh-123 efflux was measured as described by Patel *et al* [19]. Fluorescence of Rh-123 was collected through a 530/30 nm bandpass on a MoFLO high speed cell sorter (Beckman Coulter, CA, USA). After gating for live cells 10,000 cells were recorded for each sample and processed by Summit software (Beckman Coulter, CA, USA).

### Co-immunoprecipitation

Approximately 3×10^6^ cells were plated in 100 mm petri plates and allowed to grow for 24 h. Cells were collected by scraping and then lysed in RIPA buffer without DTT. Equal amount of protein (400 µg) was taken and lysates were precleared with 50 µl protein A/G-plus agarose for 30 min. Fifty microgram lysates were run as input. Agarose beads were pelleted and supernatant was incubated with FASN specific antibody overnight at 4°C. Fifty microliter protein A/G-plus agarose was added in antibody-antigen complex with gentle shaking for 4 h at 4°C. Ag-Ab complexes were centrifuged at 3,000 rpm for 5 min. Pellet was washed thrice with low salt buffer (50 mM Tris Cl pH 7.5, 25 mM NaCl and 1% Triton X-100) and high salt buffer (50 mM Tris Cl pH 7.5, 500 mM NaCl and 1% Triton X-100) each. Target and its associated proteins were disrupted and resolved on 10% SDS-PAGE. The FASN and Cav-1 proteins were detected by immunoblotting.

### Statistics

Statistical comparisons were made using student`s two-tailed unpaired t test and P value <0.05 was considered statistically significant.

## Results

### Selection and Characterization of Paclitaxel-resistant Cancer Cells

**Figure 1:**
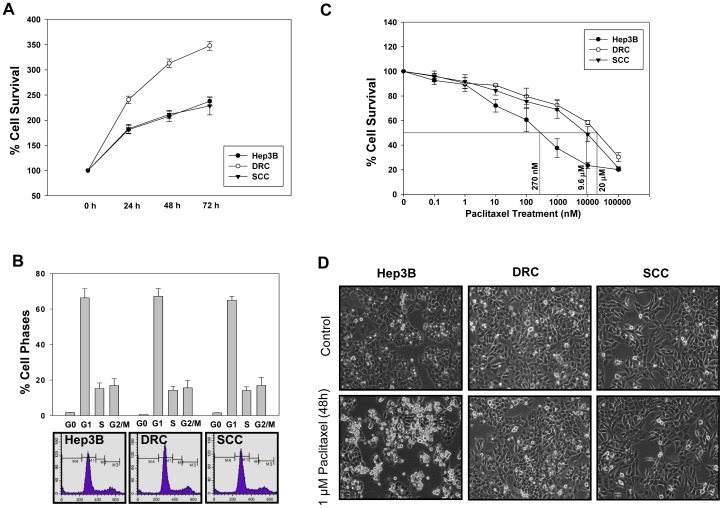
Selection and characterization of human hepatoma cell line Hep3B cells, DRC and SCC. (A) 2×10^3^ cells were plated in 96 well plates and medium was removed at indicated time interval and cell survival was evaluated by MTT assay. (B) Hep3B cells, DRC and SCC (5×10^5^) cells were plated in 35 mm petri plates and allowed to incubate for 24 h. Thereafter cell were processed for FACS analysis. (C) 8×10^3^ cells were plated in 96 well plates containing 100 µl medium. After 24 h incubation, cells were treated with varying concentrations of paclitaxel for 48 h and cell survival was evaluated by MTT assay. (D) Hep3B cells, DRC and SCC were treated with 1 µM paclitaxel for 48 h and their morphology was observed under vertical microscope and photographed.

**Figure 2:**
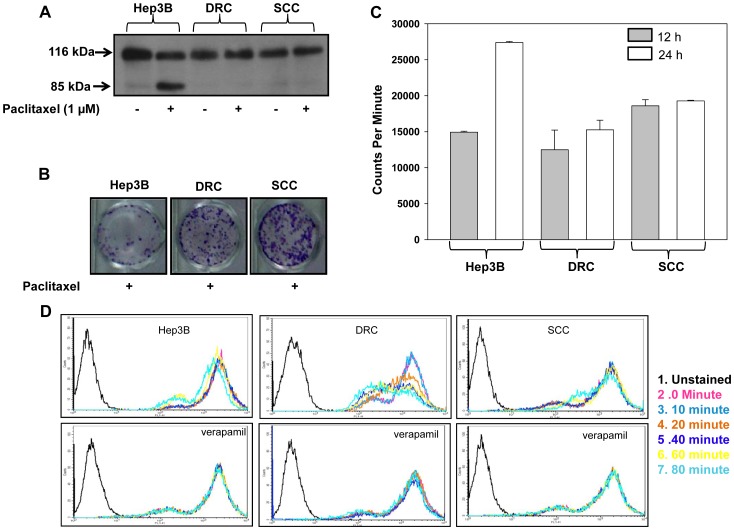
Selection and characterization of paclitaxel resistant DRC and SCC clone. (A) Hep3B cells, DRC and SCC treated with or without 1 µM paclitaxel for 48 h and PARP cleavage was analyzed by immunoblot. (B) 3×10^4^ cells were plated in 12 well plates for 24 h followed by treatment with 5.2 µM paclitaxel for 48 h. Medium was replaced with drug free medium and incubated for additional 18–20 days. Cells were then fixed and stained with crystal violet. (C) 1×10^5^ cells were plated in 12 well plates. After 24 h, ∼500 nCi/well of ^3^H paclitaxel was added to each well for 12 h and 24 h, respectively. Cells were lysed by adding SDS to respective plates. Counts were taken on Packard. (D) Hep3B cells, DRC and SCC (10^6/^ml cells) were loaded with Rhodamine-123 (2 µM) for 30 min at 37°C and efflux at respective time interval was measured by MOFLO in the presence or absence of verapamil.

Unique cellular model system to study the molecular mechanisms of acquired and inherent drug resistance was established as discussed in materials and methods (Figure S1). The cell cycle kinetics and growth curve analysis of Hep3B cells, DRC and SCC was performed. The doubling time for DRC was calculated to be 24 h and that of Hep3B cells and SCC it was 36 h. No alterations in cell cycle distribution were detected (Figure 1A and 1B). Next, to determine the IC_50_ value of paclitaxel, Hep3B cells, DRC and SCC were treated with varying concentration of paclitaxel for 48 h. IC_50_ value were calculated to be as 270 nM for Hep3B cells, 20 µM for DRC and 9.6 µM for SCC at 48 h (Figure 1C). To compare the survival capabilities of Hep3B cells, DRC and SCC were treated with 1 µM paclitaxel for 48 h. Following treatment Hep3B cells exhibited membrane blebbing and they were rounded off. On the other hand, no noticeable morphological changes were observed in DRC and SCC (Figure 1D). Further, apoptosis in these cells was determined by analyzing PARP cleavage pattern, an important marker for apoptotic cell death. No PARP cleavage was detected in paclitaxel treated DRC and SCC whereas cleaved PARP (85 kDa) was detected in paclitaxel treated Hep3B cells (Figure 2A). Additionally, long term colony formation assay was performed and it was observed that in paclitaxel treated DRC and SCC more colonies were formed compared to those in Hep3B cells (Figure 2B). The decreased sensitivity of DRC and SCC cells to paclitaxel could be due to reduced drug uptake in comparison to Hep3B cells. Therefore, we performed drug uptake assay and uptake of radioactive labeled paclitaxel [^3^H(G)] was more in Hep3B cells as compared to DRC and SCC (Figure 2C). Reduced uptake in DRC and SCC may be due to overexpression of P-gp which is responsible for effluxing of drugs from the cells. To study the involvement of P-gp in DRC and SCC, Rhodamine-123 (Rh-123) uptake, a P-gp substrate, efflux assay was performed. The peak intensity shifted from right to left (towards unstained region) in DRC indicating that Rh-123 is being effluxed out from the cells whereas no alteration in Rh-123 efflux was observed in Hep3B cells and SCC. The efflux of Rh-123 in all the three cells diminished in the presence of verapamil, and to a greater extent in DRC (Figure 2D).

### Protein Expression and Development of Resistance

**Figure 3:**
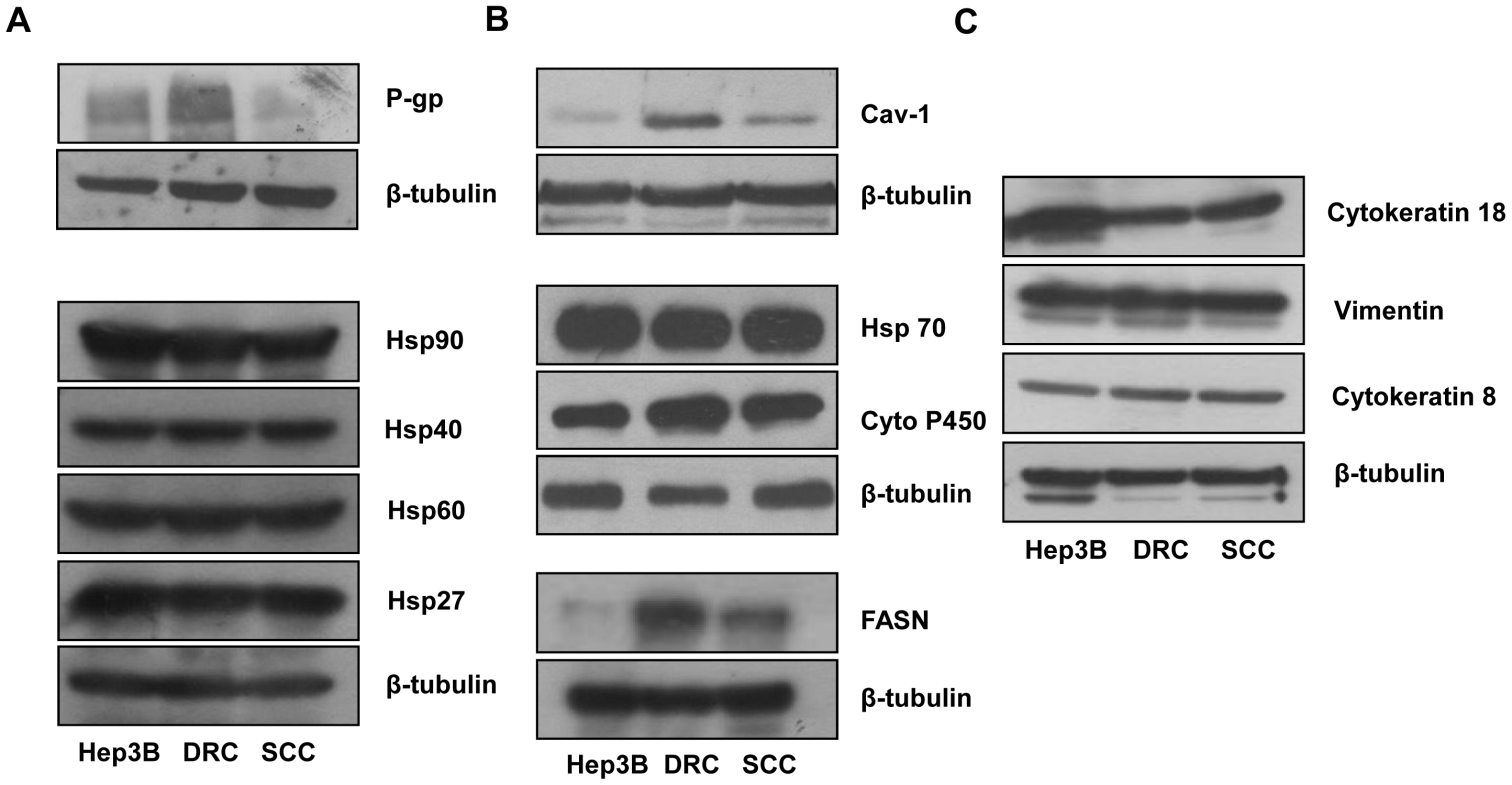
Western blot analyses of whole cell lysates of Hep3B cells, DRC and SCC showing basal level expression of various molecules. Representative western blot of basal level expression for various molecules was performed. Briefly, cells were harvested and whole cell lysates were prepared. Proteins were resolved using 8% or 10% SDS-PAGE and then processed for western blotting analysis.

Rh-123 efflux assay suggested that DRC might overexpress P-gp and to confirm this western blotting was performed. High P-gp expression was detected in DRC compared to Hep3B cells and SCC (Figure 3A). Another membrane associated protein, Cav-1 which regulates signal transduction and protein translocation in cell membrane has also been implicated in the development of drug resistance [12], [13], [20]. Additionally, Cav-1 is reported to be associated with a cytosolic protein fatty acid synthase (FASN) [17]. FASN is a multifunctional enzyme involved in the synthesis of palmitate from acetyl CoA/malonyl CoA and is reported to play a role in drug resistance in breast cancer cells [16]. Yet another molecule of importance in drug resistance is Cytochrome 450 (CYP450), an enzyme involved in biotransformation of many drugs. Metabolism by CYP450 is a major determinant in reduction of drugs induced pharmacological effect [21]. To unravel whether Cav-1, P-gp, FASN and CYP450 have a role to play in the development of resistance to paclitaxel in DRC and SCC, expression level of these molecules were examined. Cav-1 and P-gp levels are markedly increased in DRC whereas FASN and CYP450 levels increased in both DRC and SCC compared to Hep3B cells (Figure 3). Expression of Heat shock proteins (Hsps) has also been linked to the development of chemoresistance in cancer cells. For example, Hsp expression in gastric, breast and liver cancers has been correlated with the development of resistance to chemotherapy or radiation therapy [22], [23], [24]. However, no significant alteration in the levels of Hsp70, Hsp40, Hsp 90, Hsp60 and Hsp27 between Hep3B, DRC and SCC was detected (Figure 3A). Also, stem cells like property of cancer cells has been implicated in chemoresistance [25] and we did not detect any change in in the expression levels of stem cell markers vimentin, cytokeratin 8, cytokeratin 18 (Figure 3C).

### Role of P-gp and CYP450 in Acquired and Inherent Drug Resistance

**Figure 4:**
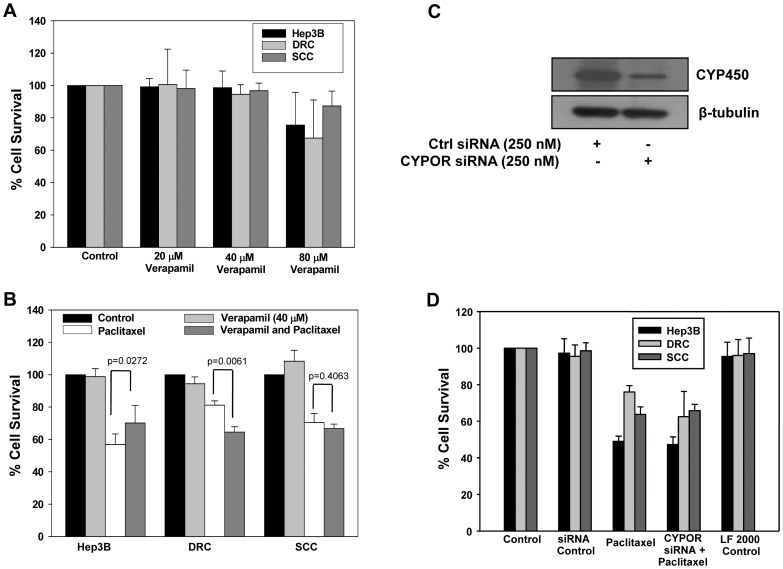
Involvement of P-gp or CYP450 in cancer drug resistance. (A) Cells were treated with varying concentration of verapamil for 24 h. Thereafter medium was replaced with fresh medium and incubated for additional 48 h. (B) Hep3B cells, DRC and SCC were pretreated with verapamil for 24 and then fresh medium was added with or without paclitaxel. After 48 h treatment, drug containing medium was removed and MTT assay was performed. (C) DRC cells were transfected with control or CYPOR siRNA as per manufacture instruction. After 24 h transfection, cell lysate was prepared and protein was resolved in 10% SDS-PAGE followed by western blotting was performed. (D) Hep3B cells, DRC and SCC were transfected with control or CYPOR siRNA. Twenty-four hours posttransfection, cells were washed and medium was replaced with fresh one with or without paclitaxel for 48 h and cell survival was evaluated by MTT assay.

P-gp level in DRC was elevated as compared to those in Hep3B cells or SCC (Figure 3A, left panel). To verify the role of P-gp, verapamil an inhibitor/blocker, was used alone or combination with paclitaxel. Upto 40 µM concentration, verapamil alone did not have any effect on the cell survival (Figure 4A). Treatment of DRC with paclitaxel in the presence of verapamil promotes cell death and cell survival reduced by 20% in DRC while no difference in cell survival was observed in SCC (Figure 4B). This suggests that apart from P-gp, other molecules may also contribute to drug resistance phenotype in DRC and SCC (Figure 4A and 4B). As CYP450 was upregulated in both DRC and SCC, we explored its involvement by siRNA mediated knockdown. The siRNA specificity was confirmed in DRC and CYP450 level was reduced by more than 3 fold (Figure 4C). Next, Hep3B cells, DRC and SCC transfected with CYP450 siRNA and treated with paclitaxel for additional 48 h. Cell survival was evaluated by MTT assay and shown in Figure 4D; knockdown of CYP450 did not have significant impact on the survival of paclitaxel treated Hep3B cells or DRC or SCC.

### Inhibiting FASN in Resistant Cells Enhances Sensitivity to Paclitaxel

**Figure 5:**
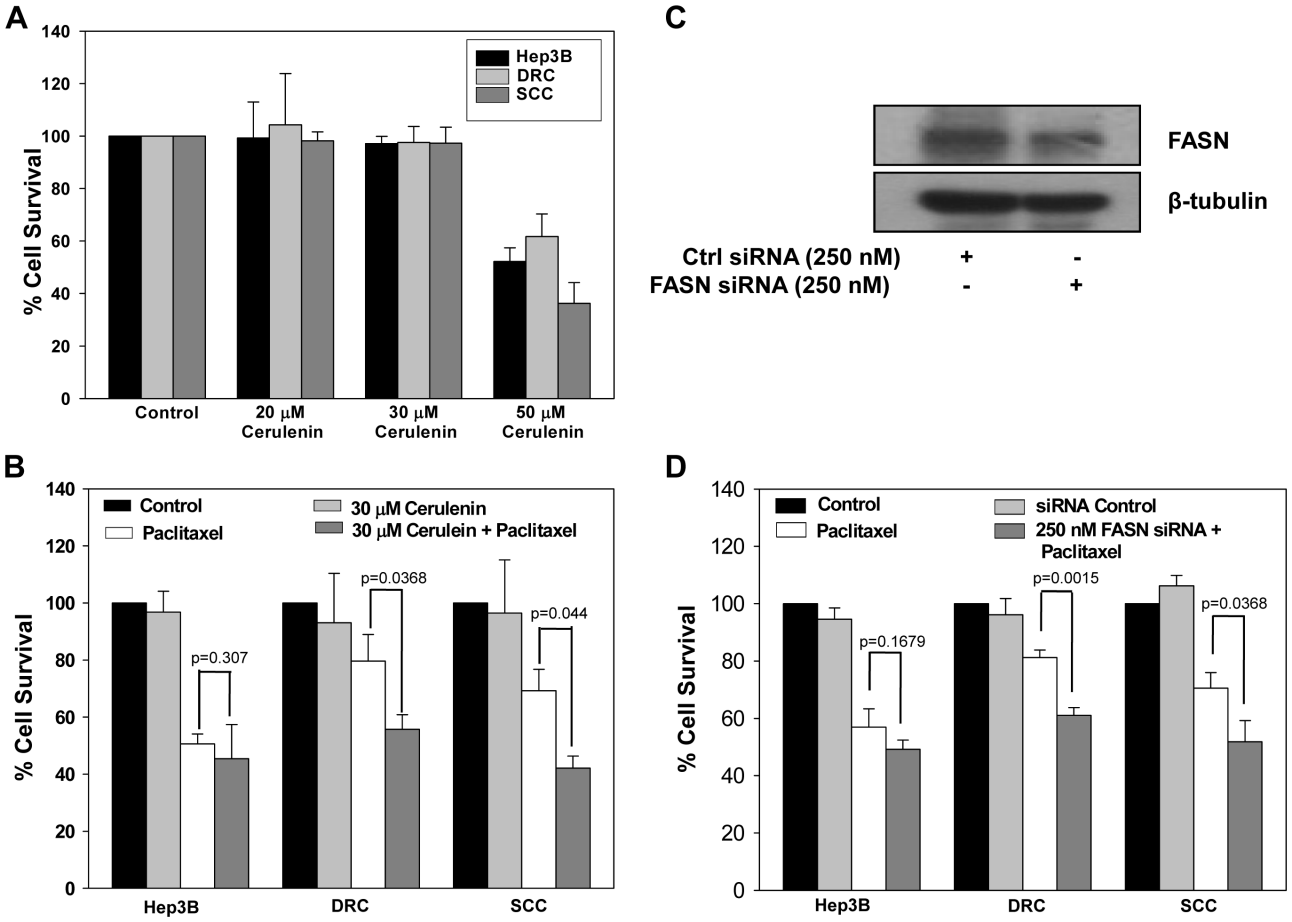
FASN knockdown sensitizes both acquired and inherent drug resistant cells. (A) Cells were treated with varying concentration of cerulenin for 24 h. Cells were washed and fresh medium was added for additional 48 h. MTT assay was performed and reading was taken at 570 nm using ELISA plate reader. (B) Hep3B cells, DRC and SCC were pretreated with 30 µM cerulenin. After 24 h treatment, medium was replaced with fresh medium with or without paclitaxel for 48 h and cell survival was evaluated by MTT assay. (C) DRC transfected with control or FASN siRNA for 24 h and western blotting was performed. (D) Hep3B cells, DRC and SCC were transfected with control and FASN siRNA as per manufacture instruction. Twenty-four hours posttransfection, medium with or without paclitaxel was added for 48 h and cell survival was evaluated by MTT assay.

FASN level correlates with tumor progression and it play an important role in tumor growth, survival and development of drug resistance [16], [17]. We found that FASN levels were elevated in DRC and SCC compared to Hep3B cells (Figure 3B). A potent inhibitor of FASN, cerulenin reduced the viability of cells in a dose dependent manner (Figure 5A). However, treatment of cells with cerulenin (30 µM) for 48 h did not have any deleterious effect on Hep3B cells, DRC or SCC survival (Figure 5A). Combination treatment of cerulenin and paclitaxel significantly inhibited cell growth in DRC (38%) as well as SCC (43%) in comparison with paclitaxel treatment alone (Figure 5B). Further, to confirm the specificity of involvement of FASN in drug sensitivity its knockdown was achieved by transfecting cells with FASN siRNA. As shown in Figure 5C, in cells transfected with specific siRNA, FASN protein levels reduced by 2 folds in comparison to control siRNA transfected cells. Next, cells transfected with FASN or control siRNA were exposed to paclitaxel for 48 h and cell survival was assessed by MTT assay. Cell survival was inhibited by 23% and 29% in DRC and SCC, respectively in comparison to control siRNA transfected and paclitaxel alone treated cells (Figure 5D).

### Downregulation of Cav-1 in DRC Re-sensitizes Cells to Paclitaxel

**Figure 6:**
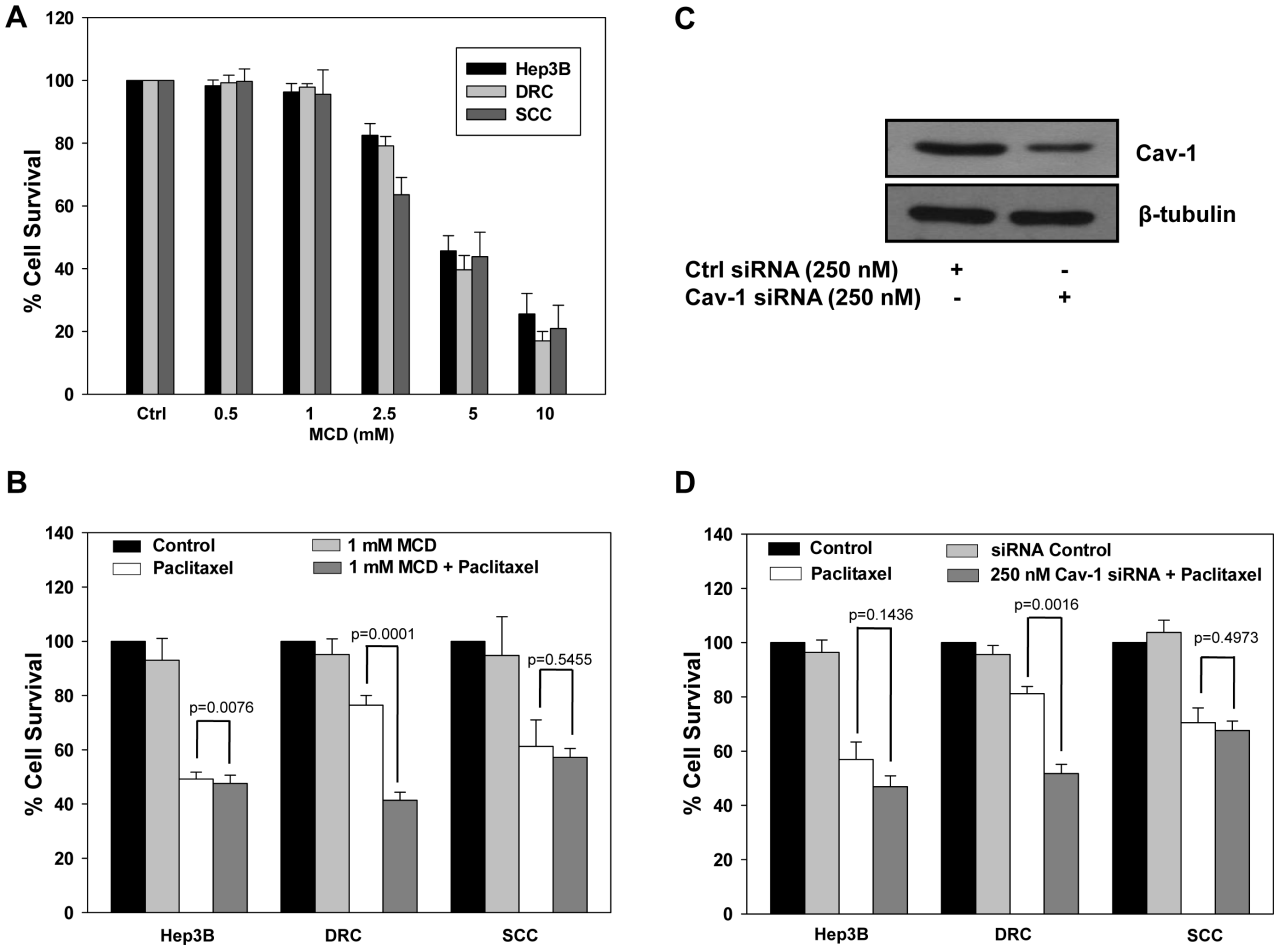
Cav-1 knockdown sensitizes acquired drug resistant clone (DRC) towards paclitaxel. (A) Hep3B cells, DRC and SCC were treated with MCD for 4 h. Thereafter, fresh medium was added for additional 48 h and cell survival was evaluated by MTT assay. (B) Hep3B cells, DRC and SCC were treated with MCD for 4 h and then fresh medium with or without 300 nM paclitaxel were added for further 48 h. After indicated treatment with or without paclitaxel, medium was removed and MTT assay was performed. (C) DRC cells were transfected with control or Cav-1 siRNA, respectively as per manufacturer instruction. After 36 h, cell lysates were prepared and Cav-1 expression was analyzed by western blotting. (D) 8x10^3^ cells were plated in 96 well plates and allowed to incubate for 24 h. Cells were transfected with control and Cav-1 siRNA for 36 h and then fresh medium containing 300 nM paclitaxel was added for additional 48 h. Thereafter cell survival was evaluated by MTT assay.

It has been reported that expression level of Cav-1 correlates with paclitaxel resistance in lung cancer cells [12], [13]. Interestingly, Cav-1 protein level was more in DRC compared to Hep3B cells and SCC (Figure 3B) and we hypothesized its role in the development of resistance. Hep3B cells, DRC and SCC when treated with increasing concentration of MCD, a non-specific inhibitor of Cav-1, cell viability decreased in a dose-dependent manner (Figure 6A). Sensitivity of all the cell lines to the combination of MCD and paclitaxel together was pronounced and the viability of DRC significantly reduced by 47% in comparison to individual treatments (Figure 6B). Under identical experimental conditions viability of Hep3B cells and SCC was unaffected. Furthermore, to ascertain the specific role of Cav-1, we knocked down Cav-1 by using siRNA and its expression decreased by 3-folds in DRC (Figure 6C). Downregulation of Cav-1 by siRNA increases the sensitivity of DRC towards paclitaxel (Figure 6D).

### Acquired and Inherent Resistant Cells Exhibit Cross Resistance Towards Other Anti-cancer Agents

**Figure 7:**
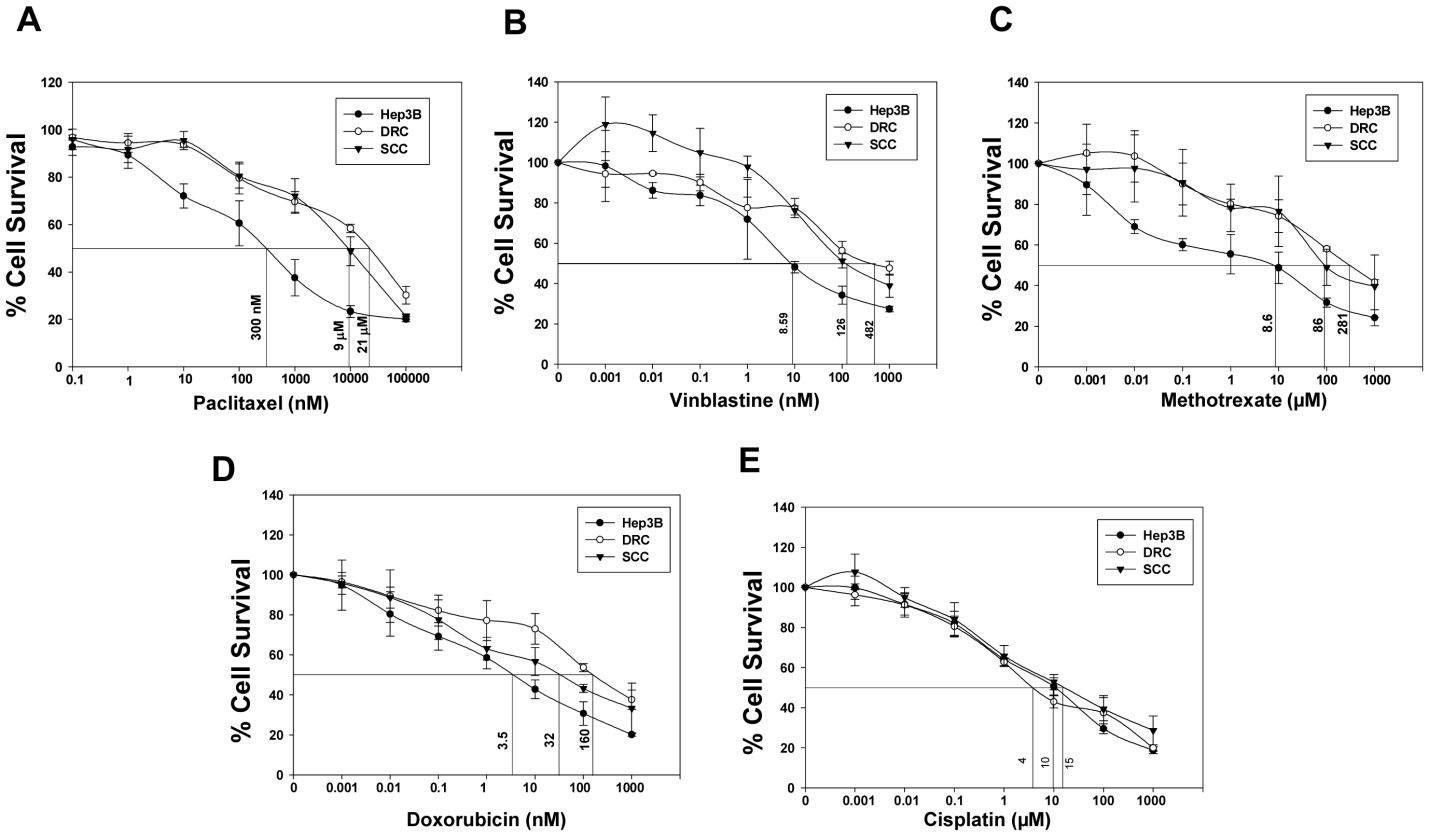
DRC and SCC exhibit cross resistance to different classes of anti-cancer agents. (A–E) Hep3B cells, DRC and SCC were plated and treated with different concentration of Paclitaxel, Vinblastine, Methotrexate, Doxorubicin and Cisplatin. After respective drug treatment for 48 h, drug containing medium was replaced and cell survival was evaluated by MTT assay.

So far we demonstrated that increased protein levels of Cav-1 or FASN or P-gp predisposes cells towards drug resistant phenotype. To explore whether levels of these proteins also predicts cell fate following treatment with other chemotherapeutic drugs, sensitivity of cells assessed by MTT assay. DRC and SCC were resistant to Vinblastine (Vin), Methotrexate (MTX), Doxorubicin (Dox) though they were equally sensitive to cisplatin (Figure 7B–E). The IC_50_ for these drugs is summarized in Table 1. The impact of downregulation of Cav-1, FASN, P-gp and CYP450 on the outcome of treatment with different drugs is compiled in Figure S2 and S3. Interestingly, knockdown of Cav-1 by siRNA followed by addition of drugs drastically decreases cell survival in DRC compared to drug alone treated cells. Downregulation of Cav-1 also affected survival of SCC treated with drugs. Knockdown of FASN by siRNA significantly reduces the survival of SCC and DRC (Figure S2). CYP450 downregulation had no effect on the survival of DRC and SCC with or without drug treatment. Interestingly, verapamil treatment diminishes cells survival to somewhat lesser extent then the levels achieved by knockdown of Cav-1 or FASN by siRNA (Figure S3).

**Table 1:** Resistance factor of DRC and SCC to anti-cancer agents.

Drugs	Hep3B (IC50)	DRC (IC50)	SCC (IC50)	DRC/Hep3B(resistance fold)	SCC/Hep3B (resistance fold)	DRC/SCC (resistance fold)
Vinblastine	8.59 nM	482 nM	126 nM	56.11	14.66	3.82
Methotraxate	8.6 µM	281 µM	86 µM	32.67	10.00	3.26
Doxorubicin	3.5 nM	160 nM	31 nM	45.71	8.85	5.16
Cisplatin	4.0 µM	15 µM	10 µM	3.75	2.50	1.50

### Inter-relationship between Cav-1, FASN, P-gp and CYP450

**Figure 8:**
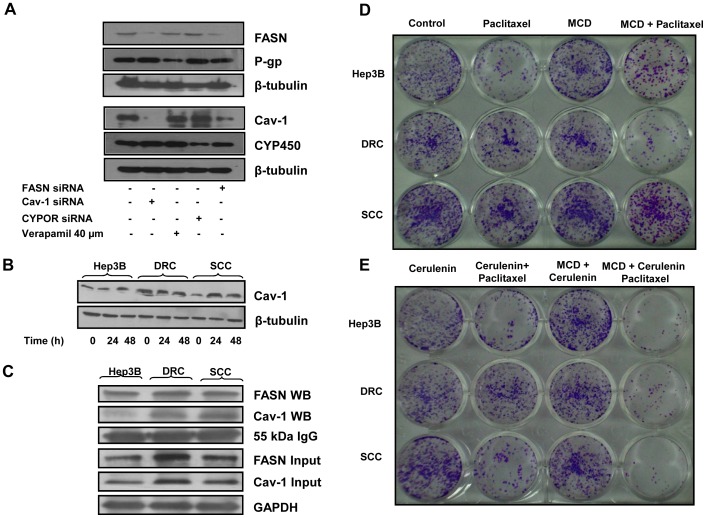
Cav-1 knockdown inhibit the expression of FASN and vice versa. (A) DRC (5×10^5^) were plated in 35 mm petri plate. After 24 h, cells were transfected with siRNAs targeting Cav-1, FASN or CYPOR as per manufacturer instruction. Simultaneously, 40 µM verapamil was added for 24 h. Thirty-six hours posttransfection or 24 h verapamil treatment, cells were harvested and lysates were prepared. Fifty microgram whole cell lysate proteins were resolved on 8% or 10% SDS-PAGE and western blot was performed. (B) Hep3B cells, DRC and SCC were treated with 300 nM paclitaxel for 24 and 48 h respectively. Whole cell lysates were prepared and 30 µg was resolved on 10% SDS-PAGE and western blotting was performed. (C) Co-immunoprecipitation of Cav-1 and FASN in Hep3B cells, DRC and SCC was carried out using FASN specific antibody. Cav-1 and FASN were detected in the immune complex by immunoblotting. IgG heavy chain and GAPDH served as loading control. (D and E) Hep3B cells, DRC and SCC were plated and allowed to adhere for 24 h and cells were pretreated with MCD (4 h) or cerulenin (24 h). After inhibitor treatment, paclitaxel was added for additional 48 h. Cells were washed with PBS, fresh medium was added and cells allowed to form colonies for ∼ 21 days. Colonies were stained with crystal violet and photographed.

It is evident from the results presented that resistant phenotype of the cancer cells is not due to alteration in only one cellular protein and other proteins may also play an important role. Therefore to better understand the inter-relationship between these molecules, we choose to look into the central role of FASN as its levels were enhanced in DRC as well as SCC. When FASN expression was knocked down by siRNA, it not only decreases FASN levels, but also diminished Cav-1 levels. Similarly, upon knock down of Cav-1 by its specific siRNA, FASN levels also decreased (Figure 8A). Knockdown of either CYP450 or P-gp did not have any impact on the expression levels of FASN or Cav-1 (Figure 8A). Interestingly, in paclitaxel treated Hep3B cells and SCC, Cav-1 level increased in a time dependent manner and in DRC no change in Cav-1 was detected (Figure 8B). To determine whether Cav-1 interacts with FASN, whole cell lysates from Hep3B cells, DRC and SCC were immunoprecipitated with FASN specific antibody and the immunoprecipated complex was then subjected to western blot analysis and probed for Cav-1 and FASN. As shown in Figure 8C, we detected Cav-1 in the immunocomplex suggesting a functional interaction between Cav-1 and FASN in HCC cells.

### MCD or Cerulenin Enhances the Effect of Paclitaxel in Long Term Survival Assay

Cerulenin or MCD enhances the cytotoxicity of paclitaxel in DRC and SCC (Figure 5, 6B). To further strengthen this observation, long term cell survival assay was performed under identical conditions. In MCD or cerulenin pretreated cells followed by paclitaxel treatment significantly reduced number of surviving colonies were detected in DRC and SCC. Additionally, we also evaluated the effect of combination of MCD and cerulenin followed by paclitaxel administration in DRC and SCC. Interestingly, number of DRC and SCC surviving colonies was significantly diminished compared to those present in cells treated with either agent alone (Figure 8D and 8E). Identical results were obtained when experiments were performed using siRNA against Cav-1 or FASN (Figure S4). To verify the role of FASN and Cav-1 in other HCC cell lines, we confirmed their expression in HepG2 and SK-HEP-1 cells (Figure S5A). Subsequently, to ascertain that these two proteins are a determinant of sensitivity to paclitaxel, we knocked down these by transfecting the cells with Cav-1 and FASN siRNA. Downregulation of FASN or Cav-1 by siRNA results in increases in the sensitivity of HepG2 and SK-HEP-1 towards paclitaxel as detected by MTT cell survival assay (Figure S5B) and as shown in long term survival assay (Figure S5C).

## Discussion

Chemotherapy is the most common treatment option for various cancers. However, its effectiveness is often compromised because of inherent or acquired drug resistance, with major impediment being frequent appearance of multi-drug resistant cancer cells during drug treatment [26]. Multiple factors may act synergistically or individually and contribute towards inherent as well as acquired chemoresistance towards a broad spectrum of anticancer drugs, eventually leading to par below expected outcome in cancer chemotherapy [27], [28], [29]. Great deal of work has been done to know the mechanisms, especially, on P-gp towards the development of resistance in cancer cells [27], [28]. Nonetheless, question remains as to why degree of resistance varies between the individuals and also for the drugs utilized. Upon exposure of tumor cells to drugs, it is likely that vulnerable cells are killed and resistant cells survive to proliferate. Additionally, these cells may exhibit resistance not only to the original drug used but also to many other chemotherapeutic drugs, a phenomenon known as cross resistance. Therefore, deciphering the factors contributing to resistant phenotype may provide therapeutic targets that in turn aid to address the resistance and restore chemosensitivity.

Use of available cell lines can address only one aspect of human drug resistance and hence disparities and misinterpretations are inevitable when a comparison is drawn between *in* *vitro* and *in vivo* observations [30]. Such drawbacks have been the major hurdles in the translation of preclinical findings to the clinic. Therefore, it is extremely important to develop a rational model that mimics some of the *in vivo* phenotype.

In the present model system, drug resistance phenotype was confirmed by detecting induction of apoptosis in parental Hep3B cells following treatment with paclitaxel whereas no PARP cleavage was detected in both SCC and DRC. Also, compared to Hep3B cells, more colonies were detected in DRC and SCC in long term survival assay in paclitaxel treated cells. Although both DRC and SCC exhibited resistance to paclitaxel, these cells significantly differed in growth properties. DRC doubled faster than parental cells and SCC. Also, the uptake of radiolabeled paclitaxel was maximum in Hep3B cells followed by SCC cells and lesser in case of DRC. Furthermore, Rh-123 was effluxed out more rapidly from DRC compared to Hep3B cells and SCC. Thus, initial characterization indicates that this cellular model mimics the general observation that tumors which become resistant to the drugs eventually grow more rapidly and eflux drugs out due to P-gp overexpression. This cellular model would therefore facilitate in further exploring the complexity to drug resistance phenomena.

First, we investigated those molecular alteration that may be playing a role. As expected, P-gp was highly unregulated in DRC compared to Hep3B cells and SCC. In addition to P-gp, Cav-1, FASN and CYP450 expression was also verified as these have been reported to be associated with the development of chemo resistance. Cav-1 is a multifunctional protein and it has received increased consideration in cancer because of its role in cell survival and in drug resistance. Drugs may down regulate or up regulate the expression of Cav-1 depending upon cell types [12], [13]. Recently, it has been reported that Cav-1 expression is correlated with HCC tumorigenic, metastasis, invasion, survival and poor prognosis [14], [15]. Cav-1 levels were increased only in DRC suggesting its association with acquired drug resistance. Increased expression of FASN has been correlated with poor prognosis and chemo resistance in breast cancer cells [16] and to our knowledge there is no report on the relevance of FASN levels in chemo sensitivity of HCC. Interestingly, FASN levels are increased in both DRC and SCC (Figure 3B).

Our data further demonstrates that drug resistant cells could be resensitized by utilizing pharmacological inhibitors or specific siRNA against these molecules reascertaining their association with the development of chemoresistance in HCCs. Downregulation of P-gp expression using verapamil decreased cell survival in DRC by ∼20% (Figure 4B) whereas knockdown of CYP450 did not significantly alter survival in any of these cells exposed to paclitaxel (Figure 4D). Thus, FASN appears to plays a central role in inherent as well as acquired resistance in HCC cells. Both FASN inhibitor cerulenin and FASN siRNA, had profound effect on the survival of paclitaxel treated DRC and SCC compared to paclitaxel treatment alone (Figure 5B and 5D). Surprisingly, knocking down of Cav-1 by its specific siRNA or by treating cells with MCD, followed by paclitaxel treatment resulted in resensitization of DRC only (Figure 6B and 6D). These results indicate that Cav-1 levels are associated with the maintenance of acquired drug resistance.

**Figure 9:**
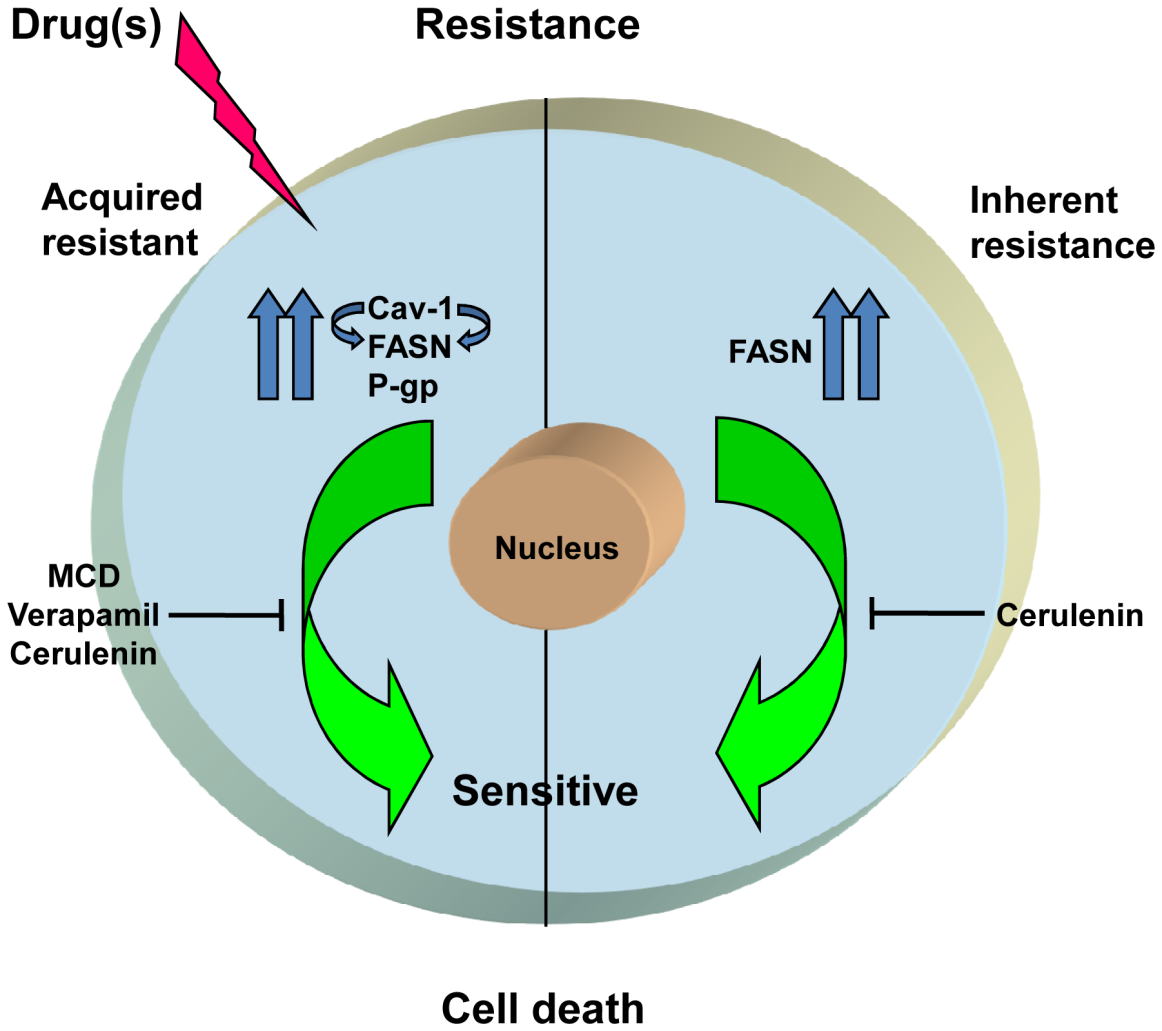
Schematic representation of proposed mechanism of drug resistance in DRC and SCC.

It has been reported that cells resistant to one drug also exhibit cross-resistance to other classes of drugs [31], [32]. Similarly, DRC and SCC exhibited resistance towards Methotrexate (MTX), Vinblastine (Vin) and Doxorubicin (Dox) (Figure 7B–D), but not against cisplatin treatment (Figure 7E). These results indicate that the factors responsible for chemo-resistance may vary depending on the class of drugs utilized. Importantly, the resistant cells could be resensitized to these different classes of drugs by silencing the protein levels of P-gp, Cav-1 and FASN, while no change in the degree of resistance was detected by abrogation of CYP450 levels. (Figure S2 and S3). Recently, it has been reported that FASN and Cav-1 interact together and modulate each other in melanoma cells [17]. Our finding that FASN and Cav-1 interact is an indicative of similarities in the modulation of FASN by Cav-1 and vice versa between HCC cells and melanoma. Finally, it can be stated that the treatment with sub-optimal levels of paclitaxel enhances Cav-1 levels which further leads to stabilization of FASN and vice versa. In addition P-gp levels are also elevated. Using inhibitors of FASN, Cav-1 and P-gp together could resensitizes resistant cells towards paclitaxel or use of FASN inhibitor alone would suffice to sensitizes the cells to paclitaxel at early stages (Figure 9).

In summary, present study highlights the fact that Cav-1 and P-gp play a crucial role in acquired drug resistance caused by paclitaxel exposure. FASN appears to be a critical factor responsible for inherent and acquired resistance towards different classes of drugs. This study provides valuable information for the improvement in chemotherapies by targeting key molecules such as Cav-1, FASN and P-gp in drug resistant HCCs. Importantly, strategies involving the use of FASN inhibitors prior to drug treatment may be preventive in transformation of inherently resistant cells to acquired drug resistance, which eventually culminates in development of multiple drug resistance phenotypes.

## Supporting Information

Figure S1 **Schematic representation of development of drug resistance cells of Hep3B cells.** Development of acquired and inherent drug resistant cells to paclitaxel as described in materials and methods.
(TIF)

Figure S2 **Knockdown of Cav-1 or FASN by siRNA decreases cell survival in DRC and SCC.** Hep3B cells, DRC and SCC (8×10^3^) were plated in 96 well plates and allowed to adhere for 24 h. Next, cells were transfected with Cav-1 or FASN siRNA as per manufacturer instruction and respective drug was added for additional 48 h. Following treatment, medium was removed and cell survival was evaluated by MTT assay.
(TIF)

Figure S3 **Verapamil along with paclitaxel treatment decreases cell survival in DRC and SCC.** Hep3B cells, DRC and SCC were plated in 96 well plates and allowed to adhere for 24 h. Subsequently cells were transfected with CYPOR siRNA or pre-treated with verapamil for 24 h and drug was added for additional 48 h. Following treatment, medium was removed and cell survival was evaluated by MTT assay.
(TIF)

Figure S4 **Knockdown of Cav-1 or FASN by siRNA followed by paclitaxel treatment decreases number of colonies in drug resistance cells.** Hep3B cells, DRC and SCC were plated and allowed to adhere for 24 h. Cells were transfected with siRNA (36 h) targeting Cav-1 or FASN, respectively. Paclitaxel was added for additional 48 h. Cells were washed with PBS, fresh medium was added and cells were allowed to form colonies for ∼ 21 days. Colonies were stained with crystal violet and photographed.
(TIF)

Figure S5 **Knockdown of Cav-1 or FASN by siRNA followed by paclitaxel treatment decreases cell survival in HepG2 and SK-HEP-1 cells.** (A) Basal level expression of FASN and Cav-1 in HepG2 and SK-HEP-1 cells by western blotting. (B) HepG2 and SK-HEP-1 (8×10^3^) cells were plated in 96 well plates and allowed to incubate for 24 h. Cells were transfected with control, Cav-1 and FASN siRNA for 36 h. Thereafter fresh medium containing 10 nM paclitaxel was added for additional 48 h. Cell survival was evaluated by MTT assay. (C) HepG2 and SK-HEP-1 (2×10^3^) cells were plated and allowed to adhere for 24 h. Cells were transfected with control or Cav-1 or FASN siRNA for 36 h. Thereafter fresh medium containing 10 nM paclitaxel was added for additional 48 h. Cells were washed with PBS, fresh medium was added and cells were allowed to form colonies for ∼ 21 days. Colonies were stained with crystal violet and photographed.
(TIF)
