# Supplementary figures and images for: Inherent and Acquired Resistance to Paclitaxel in Hepatocellular Carcinoma: Molecular Events Involved
Source: PLoS One. 2013 Apr 16;8(4):e61524. doi: 10.1371/journal.pone.0061524 (PMC3629035; doi:10.1371/journal.pone.0061524)

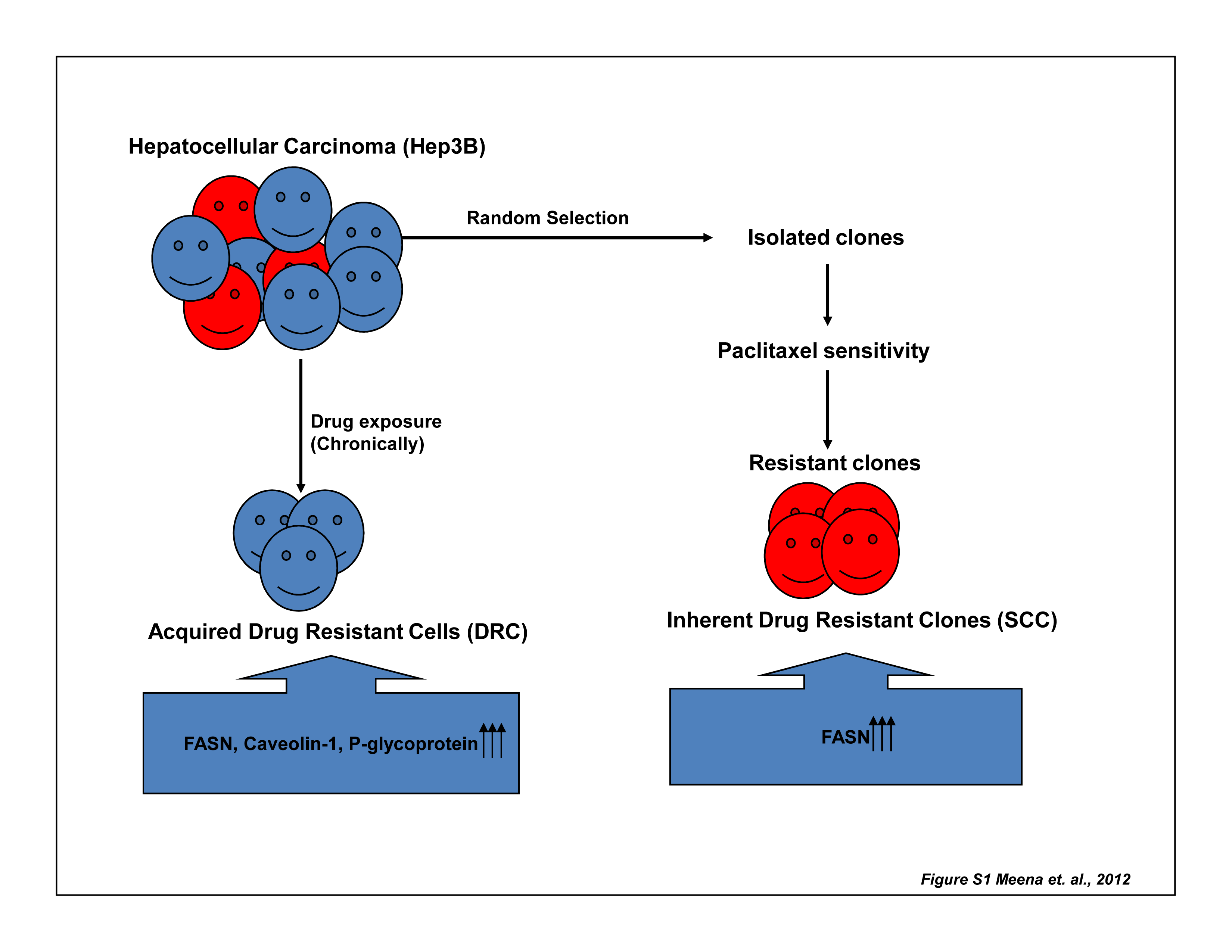

Supplement: Figure S1 — Schematic representation of development of drug resistance cells of Hep3B cells. Development of acquired and inherent drug resistant cells to paclitaxel as described in materials and methods. (TIF) [file pone.0061524.s001.tif]

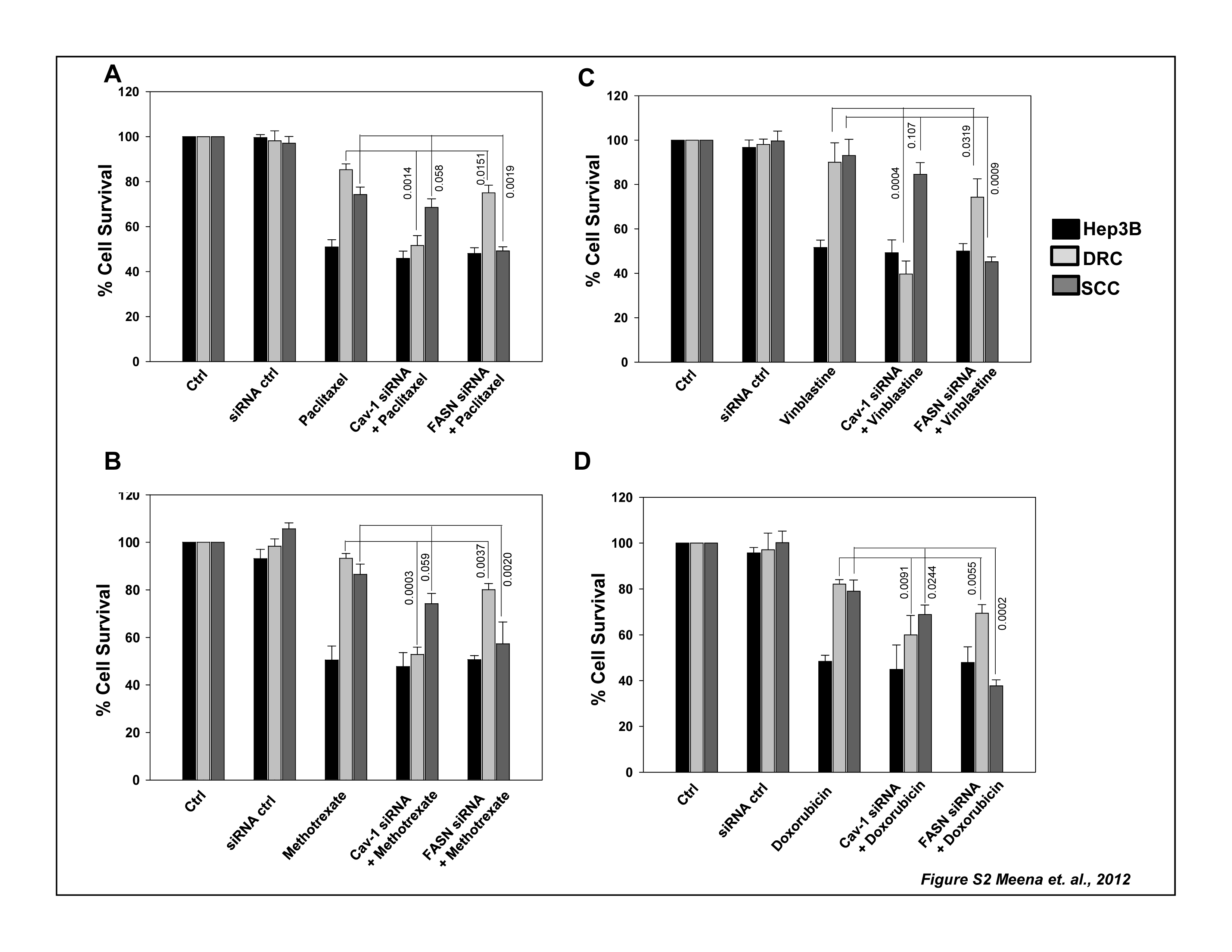

Supplement: Figure S2 — Knockdown of Cav-1 or FASN by siRNA decreases cell survival in DRC and SCC. Hep3B cells, DRC and SCC (8×10^3^) were plated in 96 well plates and allowed to adhere for 24 h. Next, cells were transfected with Cav-1 or FASN siRNA as per manufacturer instruction and respective drug was added for additional 48 h. Following treatment, medium was removed and cell survival was evaluated by MTT assay. (TIF) [file pone.0061524.s002.tif]

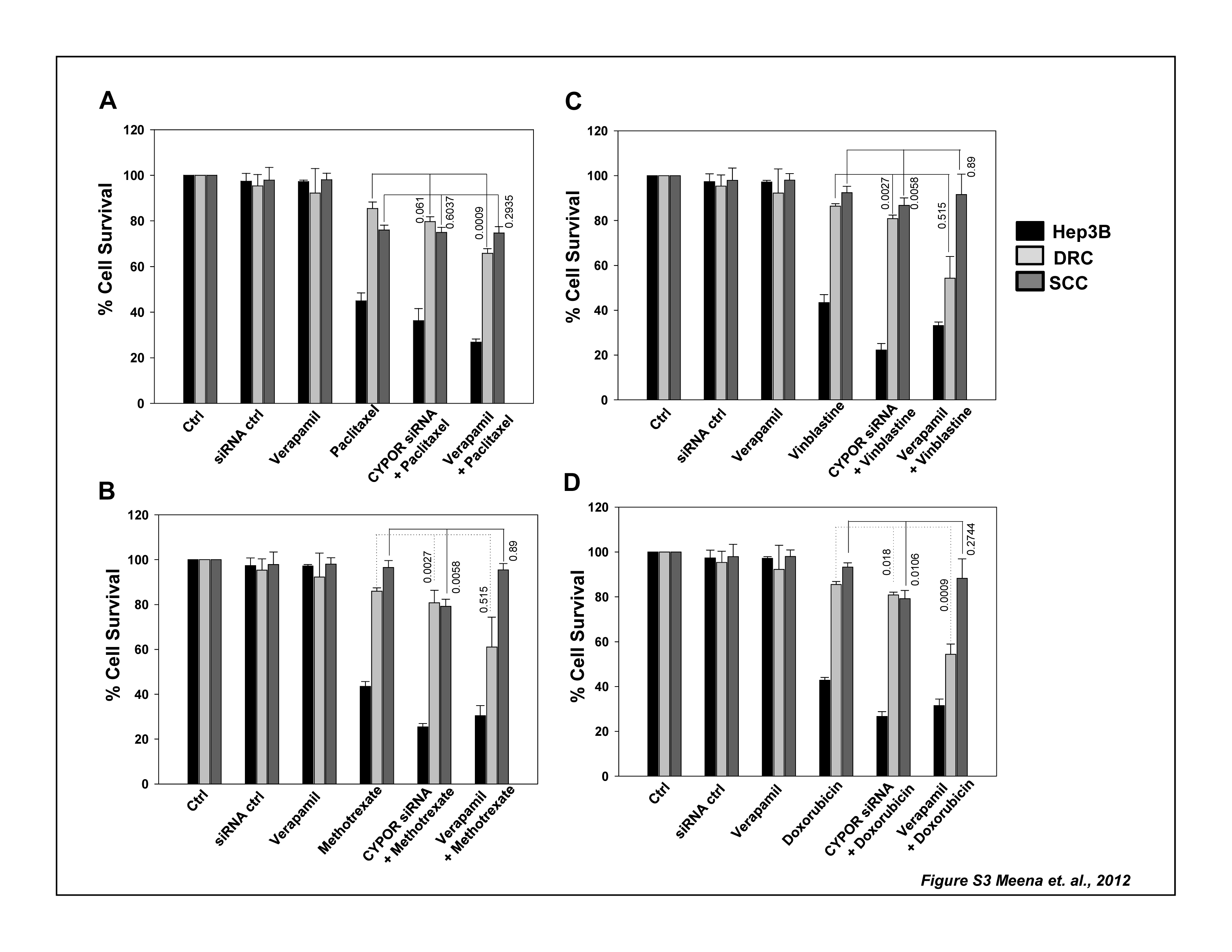

Supplement: Figure S3 — Verapamil along with paclitaxel treatment decreases cell survival in DRC and SCC. Hep3B cells, DRC and SCC were plated in 96 well plates and allowed to adhere for 24 h. Subsequently cells were transfected with CYPOR siRNA or pre-treated with verapamil for 24 h and drug was added for additional 48 h. Following treatment, medium was removed and cell survival was evaluated by MTT assay. (TIF) [file pone.0061524.s003.tif]

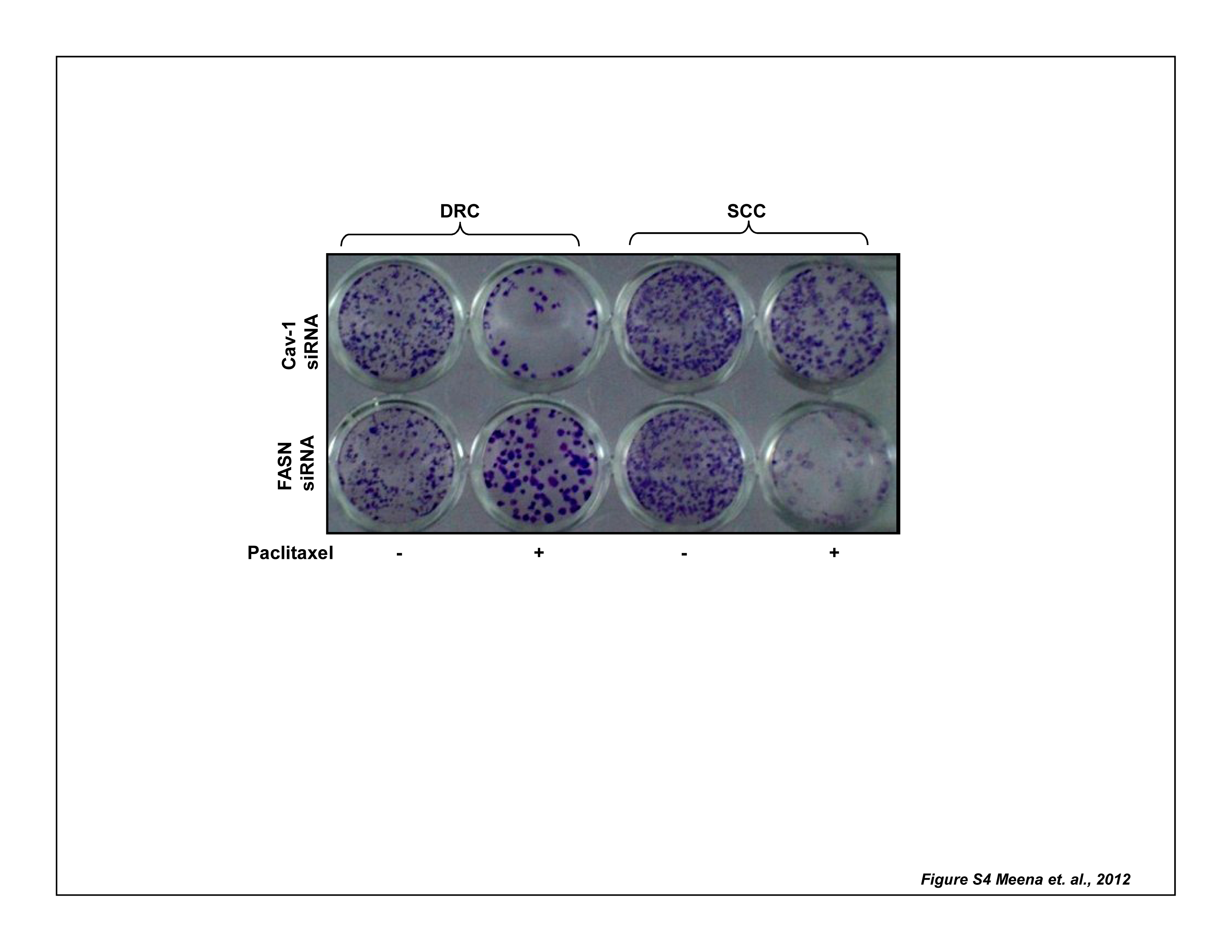

Supplement: Figure S4 — Knockdown of Cav-1 or FASN by siRNA followed by paclitaxel treatment decreases number of colonies in drug resistance cells. Hep3B cells, DRC and SCC were plated and allowed to adhere for 24 h. Cells were transfected with siRNA (36 h) targeting Cav-1 or FASN, respectively. Paclitaxel was added for additional 48 h. Cells were washed with PBS, fresh medium was added and cells were allowed to form colonies for ∼ 21 days. Colonies were stained with crystal violet and photographed. (TIF) [file pone.0061524.s004.tif]

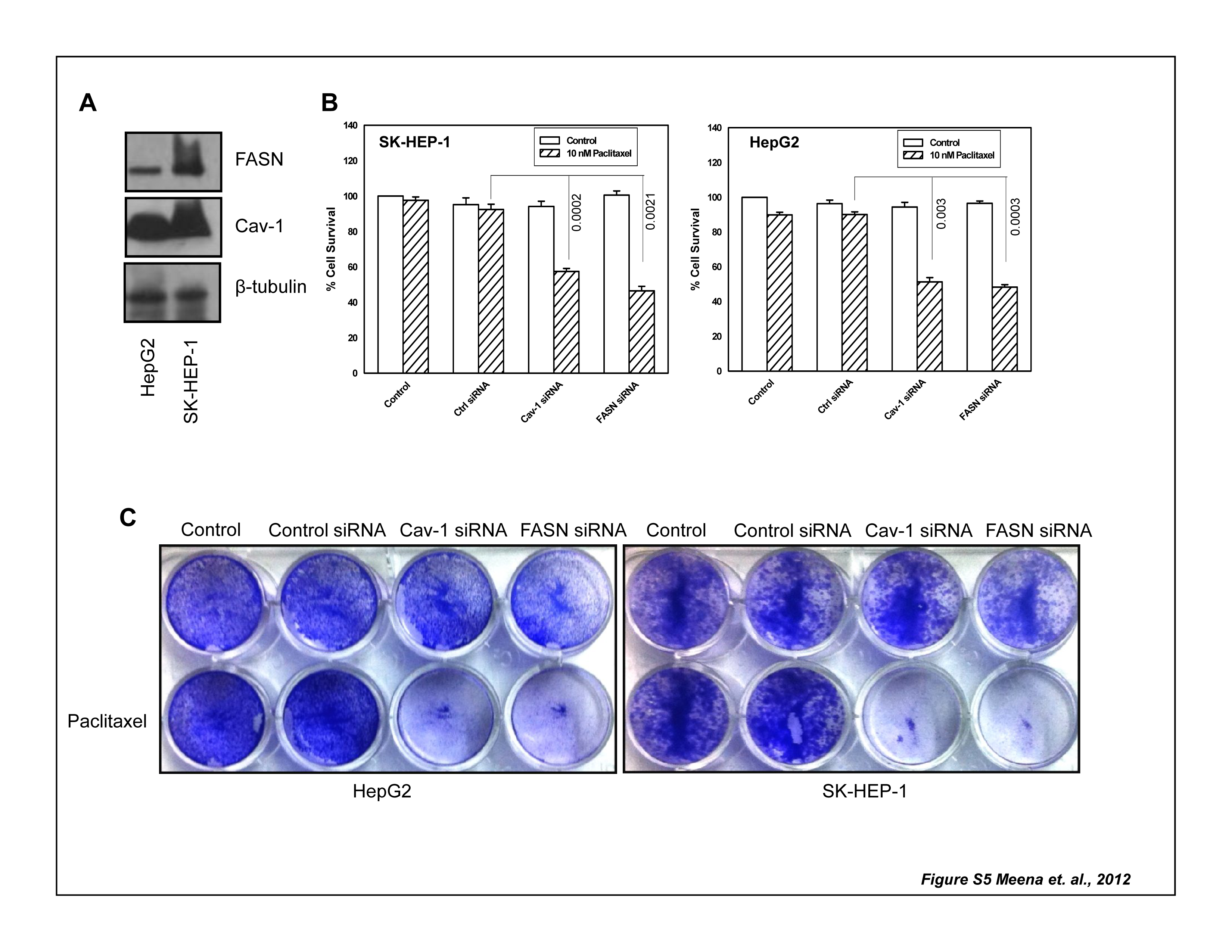

Supplement: Figure S5 — Knockdown of Cav-1 or FASN by siRNA followed by paclitaxel treatment decreases cell survival in HepG2 and SK-HEP-1 cells. (A) Basal level expression of FASN and Cav-1 in HepG2 and SK-HEP-1 cells by western blotting. (B) HepG2 and SK-HEP-1 (8×10^3^) cells were plated in 96 well plates and allowed to incubate for 24 h. Cells were transfected with control, Cav-1 and FASN siRNA for 36 h. Thereafter fresh medium containing 10 nM paclitaxel was added for additional 48 h. Cell survival was evaluated by MTT assay. (C) HepG2 and SK-HEP-1 (2×10^3^) cells were plated and allowed to adhere for 24 h. Cells were transfected with control or Cav-1 or FASN siRNA for 36 h. Thereafter fresh medium containing 10 nM paclitaxel was added for additional 48 h. Cells were washed with PBS, fresh medium was added and cells were allowed to form colonies for ∼ 21 days. Colonies were stained with crystal violet and photographed. (TIF) [file pone.0061524.s005.tif]
